# Clinical Significance of Biomarkers in Oropharyngeal Squamous Cell Carcinoma: Recurrence Prediction and Treatment Response

**DOI:** 10.1002/cnr2.70539

**Published:** 2026-04-28

**Authors:** Yunxia Chen, Wenyu Zhang, Xiang Gao, Kongling Xing, Yijing Ren, Jinyu Hu, Zhihao Xie, Ping Zhou

**Affiliations:** ^1^ Second Ward, Department of Radiotherapy The First Affiliated Hospital of Hainan Medical University Haikou China; ^2^ Department of Gastroenterology Affiliated Hospital of Jiangsu University Zhenjiang China

**Keywords:** biomarkers, liquid biopsy, oropharyngeal squamous cell carcinoma, precision oncology, recurrence, treatment response

## Abstract

**Background:**

Oropharyngeal squamous cell carcinoma (OPSCC) accounts for a substantial proportion of head and neck cancers, with a rising incidence largely driven by human papillomavirus (HPV) infection. Despite advances in multimodal treatment, disease recurrence remains common and limits long‐term survival, highlighting the need for reliable biomarkers to guide prognosis and treatment.

**Methods:**

This review summarizes recent advances in biomarker development in OPSCC across multiple biological domains. We examined molecular biomarkers, including genomic alterations, DNA methylation, and non‐coding RNAs; protein biomarkers associated with oncogenic signaling, cell‐cycle regulation, apoptosis, inflammation, and angiogenesis; as well as circulating biomarkers such as circulating tumor DNA (ctDNA), circulating tumor cells (CTCs), and exosome‐derived RNA.

**Results:**

Among currently available biomarkers, p16, PD‐L1, and circulating HPV DNA demonstrate the strongest clinical applicability, particularly for risk stratification and post‐treatment surveillance. Emerging evidence also supports the use of combined biomarker panels to improve prediction of treatment response to radiotherapy, chemotherapy, and immunotherapy. However, many candidate biomarkers show inconsistent performance due to methodological variability, limited sensitivity and specificity, and insufficient prospective validation.

**Conclusions:**

While several biomarkers show promise in OPSCC, further standardization of detection methods and large‐scale prospective studies are required. Integration of multi‐omics data with computational approaches, including artificial intelligence, may facilitate the development of robust and clinically actionable predictive models, ultimately enabling more personalized management and earlier detection of recurrence.

## 1 Introduction

Oropharyngeal squamous cell carcinoma (OPSCC) represents a major histological subtype of head and neck squamous cell carcinoma (HNSCC), accounting for ~10%–25% of all head and neck malignancies and up to 30% in certain populations [1, 2]. Squamous cell carcinoma constitutes the vast majority of cases, representing nearly 80%–90% of OPSCC. Over the past two decades, the incidence of OPSCC has risen steadily worldwide, a trend largely attributable to the dual influence of persistent tobacco and alcohol consumption and the growing prevalence of infection with high‐risk human papillomavirus (HPV), particularly HPV16 [3, 4, 5]. This shift has been especially pronounced in Western countries, where HPV‐positive OPSCC has largely supplanted tobacco‐associated disease, but similar trends are now being observed worldwide. Importantly, OPSCC is increasingly diagnosed in younger individuals and women, highlighting its evolving demographic profile and growing public health relevance. The stage at diagnosis remains a critical determinant of survival. Approximately 70% of OPSCC cases are identified at advanced stages, reducing the five‐year survival rate from 83.7% in localized disease to only 38.5% in metastatic cases [6]. The clinical landscape of OPSCC has further changed with the predominance of HPV‐positive tumors, which typically occur in younger patients with limited smoking histories and are associated with more favorable outcomes compared with HPV‐negative disease. Nevertheless, substantial heterogeneity persists within both HPV‐positive and HPV‐negative subgroups, reflecting the influence of molecular features, smoking exposure, and host‐related factors on treatment response and survival.

Current management of OPSCC relies on multidisciplinary approaches tailored to disease stage and patient characteristics. Minimally invasive transoral techniques, particularly transoral robotic surgery (TORS), have demonstrated excellent oncologic control for early‐stage disease while preserving speech and swallowing function [7]. For locally advanced cases, chemoradiotherapy remains the cornerstone of therapy. Yet these modalities are frequently accompanied by substantial acute and chronic toxicities, including xerostomia, dysphagia, and long‐term deterioration in quality of life. Even among HPV‐positive tumors, which are often more sensitive to radiotherapy and chemotherapy, the challenge of balancing therapeutic efficacy against treatment‐related morbidity remains unresolved. The concept of treatment de‐escalation has therefore been actively investigated in clinical trials, but a consensus on its safety and applicability has not yet been reached [8]. Thus, optimizing treatment intensity while minimizing morbidity continues to represent a central challenge in OPSCC care. Recurrence remains a critical determinant of long‐term outcomes. Even in HPV‐positive cohorts, ~20%–25% of patients experience recurrence within 5 years of definitive therapy [9]. Recurrent OPSCC is associated with limited salvage options, a higher risk of distant metastasis, and markedly reduced survival. Beyond its impact on mortality, recurrence imposes substantial functional and psychosocial burdens, including impaired swallowing, speech dysfunction, and diminished quality of life [10, 11, 12, 13]. These observations underscore the urgent need for more accurate tools to identify patients at high risk of recurrence and to monitor treatment response in a timely and reliable manner.

**FIGURE 1:**
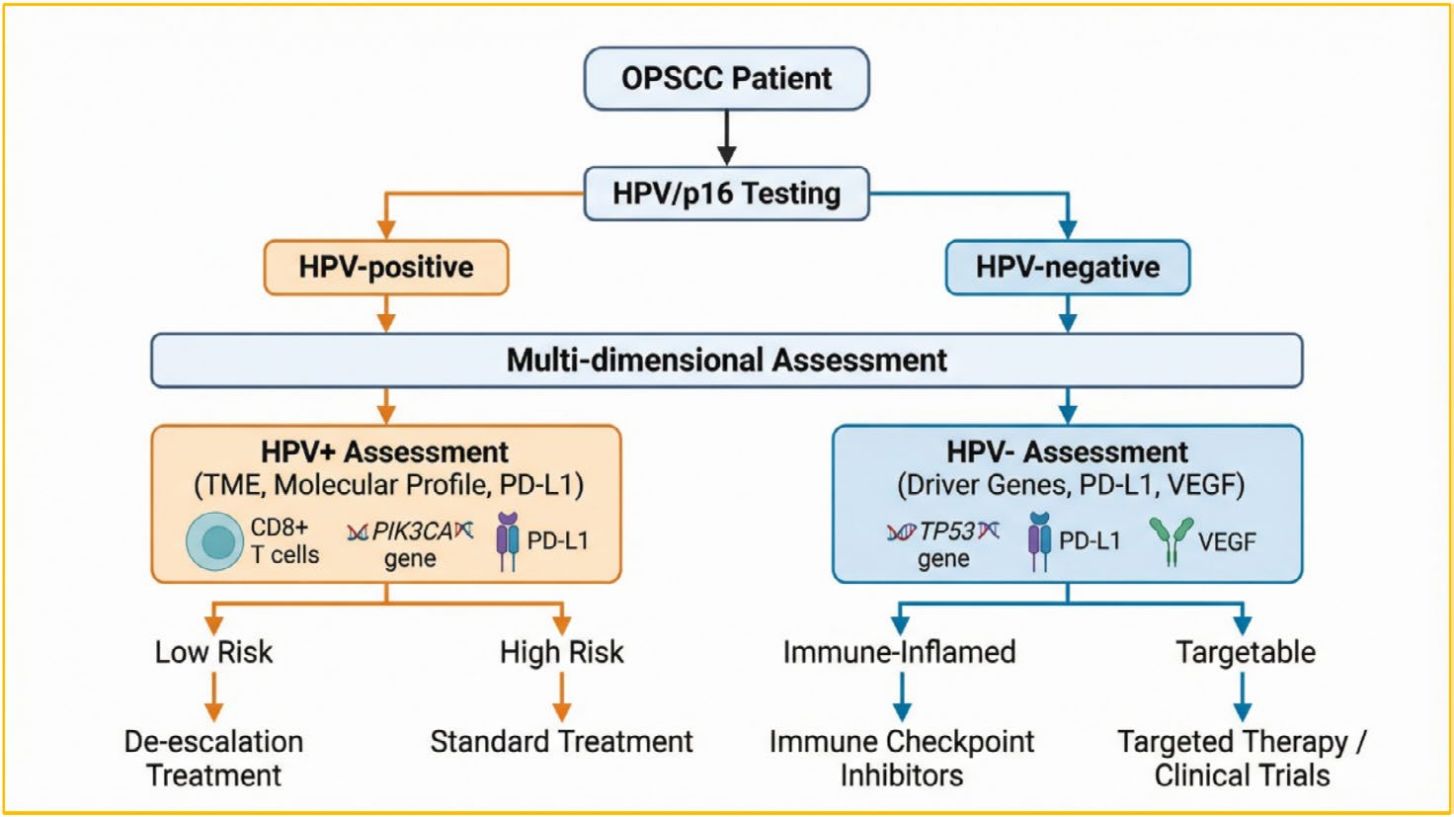
Biomarker‐driven clinical decision pathway for oropharyngeal squamous cell carcinoma (OPSCC). This schematic outlines a precision medicine framework based on HPV status. Initial HPV/p16 testing stratifies patients into HPV‐positive (orange) and HPV‐negative (blue) pathways. Each pathway incorporates key biomarkers (e.g., TME features, driver mutations, PD‐L1) to guide risk/subtype stratification, ultimately directing personalized therapeutic strategies, including treatment de‐escalation, intensification, immunotherapy, or targeted therapy. The model highlights the integration of biomarkers in optimizing OPSCC management. (Created by the authors).

Traditional approaches for treatment response assessment and surveillance, such as imaging and clinical examination, are limited by their relatively low sensitivity and temporal lag, which hinder the timely detection of tumor progression. In this context, biomarkers have emerged as a promising means to refine recurrence prediction and therapeutic monitoring. Broadly defined, biomarkers are measurable indicators that objectively reflect physiological or pathological processes or responses to therapeutic interventions. They may be derived from tissue specimens or body fluids, including blood, saliva, and urine. In oncology, biomarkers are increasingly employed in early diagnosis, disease classification, recurrence prediction, treatment monitoring, and the formulation of personalized therapeutic strategies [14, 15, 16]. With rapid advances in molecular biology and high‐throughput “omics” technologies, a growing spectrum of OPSCC‐associated biomarkers has been identified, including genetic and epigenetic alterations, protein expression profiles, and circulating tumor‐derived components. As shown in Figure 1, these biomarkers can be integrated into a comprehensive framework to support clinical decision‐making in OPSCC.

However, despite their considerable promise, several challenges currently limit the clinical translation of biomarkers in OPSCC. Many candidate markers lack standardized detection methods and validated cutoff values, and most have not yet been evaluated in large, prospective, multicenter cohorts. In addition, tumor heterogeneity and the dynamic nature of biomarker expression complicate their interpretation and longitudinal application. Addressing these limitations will be essential for integrating biomarker‐based strategies into routine clinical practice.

Although this review focuses primarily on OPSCC, much of the available molecular and clinical evidence originates from broader HNSCC cohorts, in which OPSCC represents a major biological and clinical subset. To ensure transparency and reproducibility, the literature search was conducted across PubMed, Web of Science, and Scopus using keywords including ‘oropharyngeal squamous cell carcinoma’, ‘biomarker’, ‘recurrence’, ‘chemotherapy’, ‘radiotherapy’, and ‘treatment response’. Inclusion criteria encompassed peer‐reviewed original research and reviews published in English up to December 2025, focusing on molecular, histological, and circulating biomarkers relevant to OPSCC. Studies were excluded if they did not report clinical outcomes or were limited to non‐human models. Given the substantial overlap in molecular pathways across anatomic subsites, findings from HNSCC studies remain highly relevant to OPSCC. Accordingly, this review integrates both OPSCC‐specific and selected HNSCC data to provide a comprehensive and systematic overview of current biomarkers for recurrence prediction and treatment‐response assessment, while highlighting key knowledge gaps and future directions for translational research.

## 2 Types of Biomarkers

Biomarkers are generally defined as quantifiable indicators that objectively capture normal physiological processes, pathological alterations, or the biological effects of therapeutic interventions. Within the field of oropharyngeal carcinoma, the repertoire of biomarkers has expanded considerably with the advent of molecular biology and high‐throughput technologies. Their source and mode of detection largely determine both their clinical applicability and prognostic value.

For analytical clarity, biomarkers are commonly classified into several overarching categories: tissue‐derived molecular and protein markers, circulating biomarkers detectable in bodily fluids, and functional biomarkers that reflect tumor behavior in vivo. This classification provides a useful conceptual framework through which molecular mechanisms may be systematically linked to clinical outcomes, thereby facilitating both recurrence prediction and treatment‐response assessment.

### 2.1 Tissue‐Derived Molecular and Protein Biomarkers

Analysis of tumor tissue obtained from surgical specimens or diagnostic biopsies provides direct access to genetic and molecular alterations involved in the initiation and progression of oropharyngeal carcinoma. Recurrent abnormalities, including mutations in TP53 and PIK3CA, dysregulated microRNA expression, and aberrant promoter methylation of tumor suppressor genes, have been associated with prognosis and recurrence risk. When combined with conventional histopathology, these tissue‐based markers support molecular subtyping, prognostic stratification, and treatment selection. The major advantage of tissue‐derived biomarkers lies in their high analytical specificity. However, their clinical applicability is limited by the invasive nature of tissue acquisition and the inability to perform repeated sampling for longitudinal disease monitoring. These limitations have prompted growing interest in minimally invasive circulating biomarkers as complementary tools for dynamic tumor assessment.

### 2.2 Circulating Biomarkers

Circulating biomarkers, detectable in blood and other body fluids, such as saliva and urine, have emerged as a compelling complement to tissue‐based indicators. These markers encompass circulating tumor DNA (ctDNA), circulating tumor cells (CTCs), extracellular vesicles such as exosomes, and multiple classes of non‐coding RNAs. Their principal advantage lies in the feasibility of serial sampling, enabling real‐time evaluation of tumor burden and molecular evolution.

Accumulating evidence indicates that ctDNA mirrors tumor‐specific genetic and epigenetic alterations and may facilitate early detection of recurrence and monitoring of treatment response [17]. Similarly, increased CTC counts and distinct phenotypic profiles have been associated with unfavorable prognosis and higher relapse risk. Exosomes, which carry bioactive proteins and nucleic acids, further reflect tumor–microenvironment interactions and hold diagnostic and prognostic promise. Despite these advantages, several barriers remain, including limited sensitivity in minimal residual disease, lack of standardized detection platforms, and the absence of validated cutoff thresholds. These challenges currently restrict the widespread clinical adoption of circulating biomarkers in OPSCC surveillance.

### 2.3 Protein‐ and Metabolite‐Based Biomarkers in Body Fluids

In addition to nucleic acid–based indicators, proteins and metabolites in body fluids represent an additional class of candidate biomarkers. Serum inflammatory mediators, salivary proteomic signatures, and urinary metabolomic profiles have been investigated for their potential roles in early diagnosis and recurrence monitoring. For example, elevated circulating inflammatory proteins and the detection of tumor‐associated molecules in saliva have been proposed as auxiliary tools for disease surveillance [18]. These biomarkers offer practical advantages owing to their accessibility and compatibility with routine laboratory testing. However, their clinical value remains constrained by variable sensitivity and specificity, and most candidates lack robust prospective validation. Further large‐scale studies will be required before protein‐ and metabolite‐based biomarkers can be reliably incorporated into clinical practice.

### 2.4 Functional and Immune‐Related Biomarkers

With the advent and widespread adoption of immunotherapy, increasing attention has been directed toward biomarkers that characterize the tumor immune microenvironment. Markers such as programmed cell death protein 1 (PD‐1) and programmed death‐ligand 1 (PD‐L1) expression levels, the density and composition of tumor‐infiltrating lymphocytes (TILs), and the expression of specific immune mediators have all been investigated as potential indicators for predicting treatment response and recurrence risk. These immune‐related biomarkers hold particular clinical significance in the era of immunotherapy, where they not only aid in patient stratification but also provide insights into mechanisms of immune evasion and therapeutic resistance. However, their broader clinical implementation remains hindered by the lack of standardized detection methods and interpretation criteria, underscoring the need for consensus guidelines and multicenter validation. The main categories of biomarkers and their clinical characteristics are summarized in Table 1.

**TABLE 1:** Types of biomarkers in oropharyngeal cancer and their clinical characteristics.

Type	Source	Representative examples	Clinical advantages	Limitations	References
Tissue‐based molecular and protein biomarkers	Tumor tissue (surgical specimens or biopsies)	Gene mutations (TP53, PIK3CA), DNA methylation, miRNA, abnormal protein expression	High specificity, reveal molecular mechanisms	Require invasive sampling, difficult for dynamic monitoring	[19, 20, 21]
Circulating biomarkers	Blood, saliva, urine	Circulating tumor cells (CTCs), circulating tumor DNA (ctDNA), exosomes, free miRNA/lncRNA	Non‐invasive, repeatable, enable dynamic monitoring of tumor burden	Sensitivity and standardization need improvement	[22]
Protein and metabolic biomarkers in body fluids	Serum, saliva, urine	Serum protein levels, salivary protein profiles, urinary metabolites	Easy to detect, suitable for clinical translation	Limited specificity, require further validation	[18]
Functional and immune‐related biomarkers	Tumor microenvironment, immune cells	PD‐1/PD‐L1 expression, tumor‐infiltrating lymphocytes (TILs), immune factor levels	Closely related to immunotherapy, useful for treatment response evaluation	Lack of standardized detection methods	[23]

*Note:* This table summarizes the main categories of biomarkers in oropharyngeal cancer, including their sources, representative examples, and clinical implications. Tissue‐based biomarkers provide high specificity but are limited by invasive sampling. Circulating and body fluid biomarkers offer non‐invasive, dynamic monitoring advantages, showing great potential in recurrence prediction and treatment evaluation. Functional and immune‐related biomarkers are particularly important in the era of immunotherapy, although the lack of standardized testing remains a challenge. (Table was generated by the authors based on literature data).

## 3 Biomarkers for Predicting Recurrence in Oropharyngeal Carcinoma

### 3.1 Molecular Biomarkers in Recurrence Prediction

Molecular biomarkers encompass genomic, transcriptomic, and epigenetic levels, including specific gene mutations, copy number variations, DNA methylation patterns, and RNA expression profiles. By reflecting the underlying molecular events driving tumor initiation and progression, these markers provide a valuable framework for early detection, recurrence risk stratification, and evaluation of therapeutic response. In OPSCC, molecular profiling not only distinguishes HPV‐positive from HPV‐negative disease but also supports the development of risk‐adapted surveillance and personalized management strategies.

Growing evidence indicates that epigenetic dysregulation, recurrent oncogenic mutations, non‐coding RNA imbalance, and defects in DNA repair pathways are closely associated with disease recurrence. Aberrant methylation of G protein–coupled receptor (GPCR) genes, mutations in TP53 and PIK3CA, and altered expression of selected microRNAs and long non‐coding RNAs (lncRNAs) have each been linked to unfavorable clinical outcomes. In parallel, dysregulation of DNA repair–related genes, such as ERCC1, has been associated with therapeutic resistance and an increased likelihood of relapse. Collectively, these findings underscore a gradual transition in recurrence prediction for OPSCC from conventional clinicopathologic assessment toward a more refined, mechanism‐based molecular risk model.

#### 3.1.1 DNA Methylation and Epigenetic Biomarkers

DNA methylation, a canonical epigenetic modification, characterized by the addition of a methyl group to cytosine residues within CpG dinucleotides, is catalyzed by DNA methyltransferases. Although this modification does not alter the underlying nucleotide sequence, it profoundly influences chromatin architecture and the binding affinity of transcription factors, ultimately regulating gene transcriptional activity. In general, hypermethylation of promoter regions is associated with gene silencing, whereas hypomethylation may aberrantly activate gene expression. During tumorigenesis and progression, abnormal DNA methylation is among the most frequent molecular alterations. Promoter hypermethylation can silence tumor suppressor genes, while global hypomethylation may promote genomic instability—both mechanisms contributing to recurrence and metastasis. Thus, DNA methylation not only represents a fundamental molecular mechanism in cancer biology but has also been widely investigated as a potential biomarker for diagnosis, prognosis, and recurrence prediction. In oropharyngeal cancer (OPC), aberrant DNA methylation has demonstrated notable clinical value. In a study of HPV‐associated OPC, Misawa et al. analyzed promoter methylation of 24 genes within the GPCR family across 85 primary tumor specimens and 8 matched ctDNA samples. They reported that hypermethylation of genes such as PTGDR1, PTGDR2, and PTGIR was significantly associated with recurrence risk, and these findings were validated in both tissue and liquid biopsy samples [19]. These results underscore the potential of GPCR gene methylation status as a predictive biomarker for recurrence in HPV‐positive OPC. In a subsequent genome‐wide methylation analysis, the same group identified additional genes, including CALML5, DNAJC5G, and LY6D, whose methylation profiles were strongly associated with recurrence. Collectively, these findings highlight the promise of DNA methylation markers in refining risk stratification and enabling dynamic monitoring of recurrence in clinical practice.

#### 3.1.2 Gene Mutations and Copy Number Variation Biomarkers

Genetic alterations, including point mutations and copy number variations (CNVs), represent pivotal molecular events driving the initiation, progression, and recurrence of oropharyngeal squamous cell carcinoma (OPSCC). Unlike epigenetic changes, which modulate gene expression without altering the DNA sequence, mutations and CNVs often lead to direct structural or functional changes in proteins, thereby influencing cellular proliferation, apoptosis, and genomic stability—mechanisms closely associated with recurrence risk.

Among the most clinically relevant alterations, TP53 mutations are highly prevalent in HPV‐negative OPSCC and are widely regarded as one of the most consequential molecular aberrations. The TP53 gene encodes the tumor suppressor protein p53, a central regulator of DNA damage response, cell‐cycle arrest, and apoptosis. Mutations frequently abrogate these protective functions, leading to genomic instability and enhanced tumor aggressiveness. Consistent with this biological role, Hong et al. reported that TP53‐mutant OPSCC was associated with significantly reduced overall and disease‐free survival, supporting its value as a prognostic marker of recurrence risk [20]. In HPV‐positive disease, PIK3CA mutations constitute one of the most frequent oncogenic events. Aberrant activation of the PI3K/AKT pathway driven by PIK3CA mutations enhances tumor growth and treatment resistance. Beaty et al. demonstrated that HPV‐associated OPSCC harboring PIK3CA mutations showed inferior disease‐free survival following de‐intensified chemoradiotherapy, highlighting its potential utility for risk stratification in this molecular subset [21].

Beyond single‐gene mutations, CNVs profiling has identified recurrent chromosomal imbalances with prognostic relevance. Gains of chromosome 3q26–28, encompassing oncogenes such as PIK3CA and SOX2, have been associated with aggressive tumor behavior, whereas deletions of 11q involving DNA repair genes including ATM correlate with genomic instability and unfavorable outcomes. These patterns provide additional layers of biological and prognostic information. Notably, integrative genomic approaches appear to outperform single‐marker analyses. Resteghini et al. showed that combined assessment of TP53 and PIK3CA alterations improved prognostic discrimination for recurrence and survival in head and neck cancers, a strategy likely applicable to OPSCC [24]. Together, mutation profiles and CNV signatures define distinct molecular subgroups with heterogeneous recurrence risks and offer a foundation for precision surveillance and individualized therapeutic planning.

#### 3.1.3 Non‐Coding RNA Biomarkers

Non‐coding RNAs (ncRNAs), which do not directly encode proteins but regulate gene expression and cellular processes through diverse molecular mechanisms, have emerged as critical regulators of tumor biology. Mounting evidence indicates that ncRNAs are intimately involved in oncogenesis, progression, and recurrence, rendering them promising predictive biomarkers in oropharyngeal squamous cell carcinoma (OPSCC).

Among them, microRNAs (miRNAs) have been extensively studied in head and neck squamous cell carcinoma (HNSCC). Aberrant overexpression of miR‐21, miR‐9, and miR‐155 has been consistently reported, where these miRNAs modulate pathways governing cell cycle progression, apoptosis, and invasion, thereby conferring aggressive tumor phenotypes and correlating with higher recurrence risk [25, 26]. Notably, Valenti et al. demonstrated that miR‐205‐5p directly suppresses DNA repair genes such as BRCA1 and RAD17, leading to genomic instability and significantly associating with local recurrence [27]. These findings underscore that dysregulated miRNA expression not only holds prognostic significance but also provides mechanistic insights into recurrence biology. In parallel, long non‐coding RNAs (lncRNAs), such as HOTAIR and MALAT1, are frequently upregulated across multiple malignancies. These transcripts contribute to tumor progression by modulating epigenetic regulation and activating oncogenic signaling pathways, thereby promoting migration, invasion, and metastasis [28, 29, 30, 31]. In HNSCC, aberrant expression of these lncRNAs has been linked to unfavorable clinical outcomes, reinforcing their candidacy as recurrence biomarkers. Collectively, the dysregulation of miRNAs and lncRNAs provides a valuable molecular framework for recurrence risk stratification in OPSCC. Beyond their prognostic role, ncRNAs also shed light on the molecular mechanisms driving relapse, and future integration of high‐throughput sequencing and multi‐omics profiling is expected to further refine their clinical utility.

#### 3.1.4 DNA Repair–Related Genes

DNA repair pathways play a pivotal role in safeguarding genomic integrity, and their dysfunction often accelerates mutational accumulation and increases tumor heterogeneity, thereby exacerbating recurrence risk. In oropharyngeal squamous cell carcinoma (OPSCC), alterations in DNA repair genes—whether through mutations, polymorphisms, or epigenetic silencing—have been increasingly recognized as key determinants of prognosis.

Deficiencies in major DNA repair genes such as BRCA1/2, ATM, and MLH1 compromise DNA damage response mechanisms, leading to genomic instability, aggressive tumor progression, and therapeutic resistance. For instance, Nogueira et al. demonstrated that polymorphisms in DNA mismatch repair pathway genes were associated with treatment response and clinical outcomes in patients with head and neck squamous cell carcinoma (HNSCC), highlighting the role of DNA repair mechanisms in prognostic stratification [32]. Similarly, Dutta et al. reported that single nucleotide polymorphisms in DNA repair genes, including XRCC1 and ERCC1, were associated with differential response to chemoradiation and recurrence risk, indicating their predictive value for treatment outcomes [33]. Emerging evidence also suggests that DNA repair deficiency may interact with non‐coding RNA dysregulation. For example, miR‐205‐5p has been shown to repress BRCA1 and RAD17 expression, thereby attenuating DNA repair efficiency, enhancing genomic instability, and increasing recurrence risk [27]. Such findings underscore the complex interplay between coding and non‐coding regulatory mechanisms in shaping tumor behavior. Taken together, abnormalities in DNA repair genes—including mutations, polymorphisms, and expression alterations—are strongly associated with OPSCC recurrence. Beyond their role as prognostic biomarkers, these molecular vulnerabilities may provide rational targets for novel therapeutic strategies, such as synthetic lethality approaches and PARP inhibitor–based regimens. Future efforts integrating DNA repair gene status with other molecular biomarkers hold promise for refining recurrence prediction and advancing precision management in OPSCC.

#### 3.1.5 HPV Integration Signatures

Human papillomavirus (HPV) integration into the host genome is widely regarded as a pivotal molecular event driving the initiation and progression of HPV‐associated oropharyngeal squamous cell carcinoma (OPSCC). In contrast to the episomal state, viral DNA integration is frequently accompanied by heightened genomic instability and the loss or inactivation of key viral regulatory genes. Of particular significance is the disruption of the E2 gene, which normally restrains the oncogenic functions of E6 and E7; its deletion results in unchecked activation of tumorigenic pathways, fostering uncontrolled cellular proliferation and therapeutic resistance.

This molecular alteration is implicated not only in tumorigenesis but also in recurrence risk. A study by Heft‐Neal et al. demonstrated that loss of HPV16 E2 in HPV‐positive head and neck squamous cell carcinoma was significantly correlated with reduced disease‐free survival (DFS) and increased cisplatin resistance, underscoring its potential role as a prognostic biomarker for recurrence prediction [34]. Beyond these clinical associations, recent mechanistic investigations have further illuminated the oncogenic potential of HPV integration. Khan et al. reported that HPV integration can directly induce host gene fusion events, such as the oncogenic FGFR3–TACC3 fusion, thereby activating potent oncogenic signaling pathways [35]. These fusion genes are highly expressed in proximity to integration breakpoints and synergize with E6/E7 activity to promote malignant progression. Importantly, integration‐driven gene fusions were found to be strongly associated with adverse clinical outcomes, suggesting their utility as biomarkers for recurrence prediction and treatment response assessment [35]. Furthermore, Campo et al. comprehensively evaluated circulating tumor HPV DNA, including ctHPV DNA and TTMV‐HPV DNA, as biomarkers for surveillance in HPV‐associated oropharyngeal carcinoma, providing an updated and robust synthesis of the current evidence supporting liquid biopsy‐based monitoring of disease recurrence [36]. In addition, Cooke et al. examined the clinical utility of pretreatment liquid biopsy and its association with clinicopathologic characteristics in patients with HPV‐associated oropharyngeal squamous cell carcinoma, offering important insights into the prognostic potential of circulating biomarkers prior to therapy initiation [37]. Taken together, the molecular features of HPV integration not only elucidate the unique oncogenic mechanisms underlying HPV‐associated OPSCC but also provide critical biological insights for recurrence risk stratification. Incorporating HPV status and integration patterns into predictive models may markedly enhance the precision of recurrence risk assessment and support more tailored follow‐up and therapeutic strategies.

#### 3.1.6 Molecular Residual Disease (MRD)

Molecular residual disease (MRD) refers to the persistence of trace tumor‐derived molecular signals detectable in body fluids, even when conventional radiologic imaging and routine pathology indicate complete remission. The principle of MRD assessment lies in the use of highly sensitive molecular technologies—such as digital PCR and next‐generation sequencing (NGS)—to identify minimal levels of circulating tumor DNA (ctDNA) or HPV DNA fragments, thereby enabling recurrence prediction at an earlier stage than is possible with traditional surveillance modalities.

In oropharyngeal cancer, MRD research has largely centered on ctDNA monitoring in HPV‐positive patients. Naegele et al. reported that persistent plasma ctDNA after surgery or chemoradiotherapy in early‐stage HPV‐positive OPSCC was strongly associated with recurrence risk [22]. Similarly, Chera et al. demonstrated that sustained or recurrent detection of plasma HPV ctDNA during follow‐up accurately predicted disease relapse, with a positive predictive value of 94% [38]. Compared with conventional imaging, MRD assessment offers several advantages, including non‐invasiveness, high sensitivity, and dynamic monitoring capability. Importantly, MRD can provide recurrence alerts several months—sometimes up to a year—before clinical or radiological evidence of disease emerges. This feature not only facilitates the early identification of high‐risk patients but also provides critical guidance for individualized surveillance strategies and timely secondary interventions. Taken together, MRD monitoring represents one of the most promising molecular biomarkers for recurrence prediction in OPSCC. With ongoing advances in detection technologies and the accumulation of large‐scale clinical validation, MRD is poised to become an integral component of recurrence risk management and personalized follow‐up in oropharyngeal cancer.

### 3.2 The Role of Protein Biomarkers in Recurrence Prediction

Protein biomarkers are defined as key proteins that exhibit aberrant expression or functional dysregulation during tumor initiation and progression. These include cell cycle regulators, signal transduction proteins, inflammatory mediators, and serum proteins. Such biomarkers provide a direct reflection of the biological states of tumor cells—namely proliferation, apoptosis, invasion, angiogenesis, and immune evasion—and are commonly assessed through immunohistochemistry, serological assays, or proteomic profiling. Compared with molecular biomarkers, protein‐based indicators are more closely aligned with cellular functions and phenotypic alterations, thereby offering significant value in recurrence risk assessment, prognostic stratification, and treatment response monitoring.

In oropharyngeal cancer, multiple classes of protein biomarkers have been implicated in recurrence prediction. Signal transduction proteins such as EGFR, p16, and p53 reveal alterations in proliferative signaling and tumor suppressive pathways. Proliferation‐ and apoptosis‐associated proteins including Ki‐67, Bcl‐2, Bax, and caspase‐3 reflect tumor growth potential and therapeutic resistance. Inflammatory cytokines and acute‐phase proteins such as IL‐6 and CRP mirror an activated inflammatory tumor microenvironment, which is strongly associated with recurrence risk. Furthermore, angiogenesis and metastasis‐related proteins including VEGF and MMP‐9 have been consistently linked with local recurrence and distant dissemination. Collectively, these protein biomarkers not only provide mechanistic insight into the recurrence biology of oropharyngeal cancer but also serve as practical tools for risk stratification. Importantly, they hold promise as potential therapeutic targets, offering opportunities to integrate biomarker‐guided individualized therapies and combined‐modality treatment strategies in clinical practice.

#### 3.2.1 HPV‐Related Antibodies

A defining molecular hallmark of HPV‐associated oropharyngeal carcinoma is the persistent expression of the viral oncoproteins E6 and E7. These proteins promote malignant transformation by degrading the tumor suppressor p53 and inactivating the Rb pathway, respectively, thereby driving cell‐cycle dysregulation and genomic instability. Given their continuous expression throughout tumor evolution, the host immune system frequently mounts antibody responses directed against E6 and E7. As such, E6/E7‐specific antibodies have emerged as serological biomarkers that not only reflect the pathogenic underpinnings of HPV‐driven oropharyngeal cancer but also hold promise for predicting recurrence risk.

Clinical follow‐up investigations provide supportive evidence for their prognostic utility. Longitudinal monitoring has shown that failure of E6/E7 antibody titers to decline after definitive therapy, or their persistent elevation during surveillance, is often associated with residual disease or impending relapse. For instance, Spector et al. demonstrated that significantly higher post‐treatment antibody levels in patients who subsequently recurred [39], and Fakhry et al. showed that sustained E6 seropositivity during follow‐up accurately predicted recurrence risk [40]. Notably, HPV‐related antibodies and circulating tumor‐derived HPV DNA (ctDNA) provide complementary prognostic information. Whereas ctDNA levels reflect real‐time tumor burden and allow dynamic monitoring of minimal residual disease, E6/E7 antibodies serve as long‐lived immunological memory markers, capturing cumulative exposure and persistent oncogenic signaling. Thus, integrating antibody profiling with ctDNA analysis may enhance the precision of recurrence risk stratification and enable more effective individualized surveillance strategies.

#### 3.2.2 Signal Transduction–Related Proteins

Aberrant expression of key signaling proteins plays a pivotal role in the initiation, progression, and recurrence of oropharyngeal squamous cell carcinoma (OPSCC). Among these, EGFR, p16, and p53 have emerged as the most extensively investigated biomarkers. Their dysregulation not only reflects the proliferative, apoptotic, invasive, and metastatic potential of tumor cells but also bears direct implications for therapeutic sensitivity, recurrence risk, and overall prognosis. Consequently, they have been widely explored as candidate biomarkers for recurrence prediction and treatment‐response assessment.

Epidermal growth factor receptor (EGFR) is one of the most thoroughly studied signaling molecules in OPSCC. Overexpression of EGFR activates downstream pathways, including the RAS/RAF/MEK/ERK and PI3K/AKT cascades, thereby driving uncontrolled cell proliferation and enhancing resistance to apoptosis. Clinical evidence has demonstrated that EGFR overexpression is strongly associated with increased tumor aggressiveness, higher rates of local recurrence, and poorer survival outcomes. In a landmark study, Ang et al. reported that elevated EGFR expression was associated with poor survival and distinct relapse patterns in advanced head and neck cancer, establishing its prognostic relevance [41]. Subsequent immunohistochemical analyses further confirmed that EGFR overexpression correlates with unfavorable clinicopathologic features in HNSCC, including OPSCC, supporting its value as a predictive biomarker [42]. In contrast, p16 serves as a well‐established surrogate marker of HPV‐driven OPSCC. Its sustained overexpression reflects E7‐mediated inactivation of the retinoblastoma (Rb) pathway, which disrupts cell‐cycle control. Clinically, p16 positivity consistently predicts increased radiosensitivity, lower recurrence risk, and improved survival. Population‐based reviews have emphasized its central role in defining HPV‐associated OPSCC biology [43]. Large‐scale collaborative studies, including the MARCH‐HPV project, have shown that p16 expression—especially when considered alongside smoking status—is a strong predictor of treatment outcomes after radiotherapy [44]. Collectively, these findings establish p16 as both a diagnostic and prognostic biomarker with high clinical relevance. By comparison, p53 abnormalities are predominantly observed in HPV‐negative OPSCC. Mutations in TP53 abrogate its tumor‐suppressive functions, leading to impaired DNA repair, defective apoptosis, and increased genomic instability. Numerous investigations have shown that aberrant p53 expression is strongly correlated with therapeutic resistance, unfavorable prognosis, and elevated recurrence risk. A systematic review and meta‐analysis confirmed that p53 mutational status is a valuable prognostic factor across HNSCC, including OPSCC [45]. Recent immunohistochemical data also revealed that p53 dysregulation significantly correlates with poor differentiation, advanced stage, and recurrence [42]. Taken together, these three proteins provide complementary insights into OPSCC biology: EGFR overexpression denotes an aggressive phenotype with heightened recurrence potential; p16 positivity reflects HPV‐associated tumors with favorable prognosis and reduced relapse risk; while p53 abnormalities are characteristic of HPV‐negative disease, indicating therapeutic resistance and high recurrence likelihood. Collectively, these biomarkers constitute a critical molecular foundation for recurrence risk stratification and the development of individualized management strategies in OPSCC.

#### 3.2.3 Serum and Plasma Soluble Proteins

Soluble proteins in serum and plasma have increasingly emerged as salient biomarkers for recurrence prediction in OPSCC. Their attractiveness stems from the noninvasive nature of sample collection and the maturity of detection techniques, such as ELISA and immunonephelometry. A growing body of evidence suggests that inflammatory cytokines, acute‐phase reactants, and proteins implicated in angiogenesis and immune regulation are closely linked to recurrence risk and disease progression.

Among these biomarkers, inflammatory cytokines have received particularly intensive scrutiny. Aberrantly elevated concentrations of IL‐6, IL‐8, and TNF‐α typically signify a persistently activated tumor‐associated inflammatory microenvironment. Such a milieu is capable of fostering angiogenesis, augmenting invasiveness, facilitating metastatic dissemination, and attenuating antitumor immune surveillance. Clinically, elevated preoperative IL‐6 has been consistently associated with unfavorable outcomes. For instance, Imai and colleagues, in a cohort of Japanese patients undergoing surgery for head and neck cancer, demonstrated that individuals with preoperative serum IL‐6 ≥ 8 pg/mL experienced significantly inferior overall survival (OS) and disease‐specific survival (DSS), with IL‐6 ≥ 8 pg/mL emerging as an independent adverse prognostic determinant in multivariate analysis [46]. Complementary retrospective and mechanistic investigations corroborate the pro‐tumorigenic role of IL‐6 in head and neck carcinogenesis [47]. IL‐6 activates the JAK/STAT3 pathway to enhance tumor cell proliferation, migration, oxidative stress tolerance, and ferroptosis resistance, as demonstrated by experimental studies and mechanistic reviews of IL‐6–mediated signaling in the tumor microenvironment [48]. IL‐8 has likewise been reported to possess prognostic relevance, although evidence of its independent predictive capacity in OPSCC remains relatively limited. In several malignancies, however, elevated IL‐8 levels have been linked to heightened risks of metastasis and recurrence. Acute‐phase reactants, particularly C‐reactive protein (CRP), constitute another widely studied category of soluble biomarkers. CRP elevation not only reflects systemic inflammation but also frequently serves as an independent prognostic indicator. Investigations in patients with head and neck or laryngeal cancers have suggested that high preoperative or treatment‐period CRP levels portend poorer outcomes, particularly with respect to progression‐free survival (PFS), disease‐free survival (DFS), and OS, although findings across cohorts have not always converged. Other soluble proteins have also attracted attention for their prognostic potential. Vascular endothelial growth factor (VEGF), a pivotal regulator of angiogenesis, has been correlated with tumor stage and recurrence risk in several studies. Matrix metalloproteinase‐9 (MMP‐9), by virtue of its role in extracellular matrix degradation, facilitates tumor invasion and metastasis; elevated levels in surgical drainage fluids or postoperative specimens have been associated with recurrence. More recently, soluble PD‐L1 (sPD‐L1) has emerged as a putative biomarker reflecting systemic immunosuppression. Elevated circulating sPD‐L1 has been linked to poor survival in several solid tumors, particularly in patients receiving immune checkpoint inhibitors, as supported by meta‐analyses in non‐small‐cell lung cancer, gastric cancer, and renal cell carcinoma [23]. Nevertheless, in the context of head and neck cancer and OPSCC, evidence for its utility as a recurrence predictor remains comparatively sparse. In summary, soluble proteins in serum and plasma hold considerable promise for recurrence prediction in OPSCC. Markers such as IL‐6, CRP, and VEGF have demonstrated significant prognostic value across multiple investigations. Yet, issues of sensitivity, specificity, and cohort heterogeneity persist, underscoring the need for validation in large‐scale, prospective, multicenter studies employing standardized detection protocols. Looking ahead, integrated predictive models that combine soluble proteins with molecular biomarkers, circulating tumor cells or DNA, and immune cell phenotypes may offer a more refined and individualized approach to recurrence risk stratification.

#### 3.2.4 Cell Proliferation and Apoptosis‐Related Proteins

Cell proliferation and apoptosis constitute two fundamental biological processes in tumorigenesis and cancer progression. Aberrant expression of proteins governing these pathways often signifies heightened tumor growth potential or resistance to therapy, thereby conferring critical prognostic significance in predicting recurrence of oropharyngeal carcinoma.

Ki‐67 remains the most widely utilized marker of cellular proliferation. Its overexpression reflects an active state of cell division and has been closely linked to poor differentiation in oral squamous cell carcinoma (OSCC), increased risk of recurrence, and unfavorable prognosis. In a recent investigation, Escobar et al. reported elevated Ki‐67 expression in OSCC compared with normal controls, with the highest levels observed in poorly differentiated tumors, supporting its role as an indicator of tumor aggressiveness and recurrence risk. Concurrently, an increased Bcl‐2/Bax ratio accompanied by reduced Bax expression reflects impaired apoptosis and is associated with enhanced tumor invasiveness [49]. This finding reinforces the prevailing view that Bcl‐2 overexpression and Bax loss facilitate tumor recurrence by suppressing apoptosis. In line with evidence from large‐scale clinical trials, combined detection of Bcl‐2 with other markers such as Ki‐67 and p53 has been shown to improve predictive accuracy for radiotherapy response and local control [50]. Caspase‐3, a central effector in the execution phase of apoptosis, has likewise been strongly implicated in recurrence risk. Cör et al. observed significantly diminished Caspase‐3 expression in poorly differentiated head and neck squamous cell carcinoma, suggesting that impaired apoptotic capacity is associated with adverse prognosis [51]. In contrast, Singh et al. studying an Indian cohort, demonstrated that Caspase‐3 overexpression correlated with metastatic potential and reduced survival in OSCC [52]. A large‐scale analysis by Liu et al. further revealed that elevated cleaved Caspase‐3 expression was associated with markedly shorter disease‐free survival, particularly in cases with concomitant lymph node metastasis [53]. Collectively, these findings highlight that aberrant expression patterns of Caspase‐3, whether downregulated or excessively activated, reflect apoptotic dysregulation in distinct clinical contexts, and that its dynamic alterations may serve as a potential serological tool for recurrence prediction in OPSCC. In summary, the abnormal expression of proliferation‐ and apoptosis‐related proteins including Ki‐67, Bcl‐2, Bax, and Caspase‐3, illuminates the molecular mechanisms underlying recurrence in oropharyngeal carcinoma through enhanced proliferation and impaired apoptosis. These biomarkers not only provide a rationale for risk stratification but also suggest potential therapeutic targets for individualized intervention.

#### 3.2.5 DNA Repair‐Related Proteins

The DNA repair system plays a pivotal role in maintaining genomic stability, and abnormalities in its associated proteins often lead to the ineffective repair of DNA damage, thus fostering the accumulation of mutations, enhanced drug resistance, and tumor recurrence. In head and neck squamous cell carcinoma (HNSCC), key members of the nucleotide excision repair (NER), base excision repair (BER), and mismatch repair (MMR) pathways have been firmly implicated in recurrence and treatment responses.

First, ERCC1, a core protein in the NER pathway, is primarily responsible for repairing DNA interstrand cross‐links induced by chemotherapy agents such as cisplatin. Clinical studies have shown that elevated ERCC1 expression is significantly associated with diminished efficacy of platinum‐based chemoradiotherapy, serving as an independent predictor of recurrence and prognosis [54]. In the oral squamous cell carcinoma subtype, high ERCC1 expression is similarly correlated with poor prognosis [55]. Furthermore, Ciaparrone et al. reported that high ERCC1 expression was associated with shorter disease‐free and overall survival in HNSCC patients treated with surgery followed by cisplatin‐based chemoradiotherapy [56]. Second, XRCC1 is a crucial member of the BER pathway, and its aberrant expression or genetic polymorphisms can impair the repair of single‐strand DNA breaks, thereby increasing genomic instability. Research by Mahjabeen et al. demonstrated a significant downregulation of XRCC1 in HNSCC patients, with low expression being strongly associated with tumor dedifferentiation and poor prognosis, suggesting that deficiencies in DNA repair mechanisms may accelerate the recurrence process [57]. The Arg399Gln polymorphism of XRCC1 has also been shown to correlate with differences in sensitivity to radiotherapy and chemotherapy, with patients harboring the unfavorable genotype being more prone to recurrence and reduced survival [58, 59]. On a broader scale, a systematic review and meta‐analysis by Yang et al., which included nearly 2000 patients, confirmed that high XRCC1 expression or the Arg399Gln polymorphism is significantly associated with poorer overall survival and progression‐free survival, further establishing its clinical relevance in predicting recurrence [60]. Clinical evidence also supports this conclusion. In a study by Ang et al., HNSCC patients receiving chemoradiotherapy with high XRCC1 protein expression exhibited significantly shorter median overall survival compared to those with low expression, suggesting that XRCC1 could serve as a vital prognostic marker for chemoradiotherapy efficacy and recurrence risk [61]. More recent studies have further reinforced the role of XRCC1 in recurrence prediction and prognostic evaluation. Ahirwar et al. found in a cohort of over 100 HNSCC patient samples that high XRCC1 expression was not only significantly associated with poor tumor differentiation, advanced‐stage disease, and lymph node metastasis, but also served as an independent risk factor for decreased overall survival. The researchers further proposed that XRCC1, as a key DNA repair molecule, may become a potential target for synthetic lethality‐based therapies in the future [62]. Finally, MLH1, an important member of the MMR pathway, plays a crucial role in maintaining genomic integrity. Its loss of expression or promoter methylation silencing leads to microsatellite instability (MSI) and impaired DNA repair efficiency, promoting mutation accumulation and recurrence. Some studies have indicated that MLH1 promoter methylation is closely associated with tumor progression in HNSCC [63]. Studies examining MMR gene‐related SNPs, including MLH1, MSH3, and EXO1, suggest that genetic variation in the MMR pathway influences response to platinum‐based chemoradiotherapy and recurrence risk in HNSCC patients [32]. Current systematic reviews of DNA damage response (DDR) mechanisms also underscore the potential role of the MMR pathway in recurrence prediction and treatment sensitivity [64]. In summary, DNA repair‐related proteins such as ERCC1, XRCC1, and MLH1 play a crucial role in recurrence by influencing tumor sensitivity to chemoradiotherapy. These proteins not only hold promise as molecular indicators for recurrence risk stratification and therapeutic efficacy prediction, but they may also provide a theoretical basis for optimizing individualized chemoradiotherapy regimens. Future large‐scale prospective studies and multi‐center validations will be key to advancing the clinical translation of these molecular biomarkers.

### 3.3 The Application of Circulating Biomarkers in Recurrence Prediction

Circulating biomarkers, which include tumor‐related components such as circulating tumor DNA (ctDNA), circulating tumor cells (CTCs), exosomes, and non‐coding RNAs, are present in bodily fluids like blood, saliva, and urine. Compared to traditional tissue biopsy, the detection of circulating biomarkers offers advantages such as being non‐invasive, repeatable, and capable of dynamic monitoring. These biomarkers reflect the overall molecular characteristics and evolutionary processes of tumors, thus demonstrating unique value in predicting the recurrence of oropharyngeal cancer.

#### 3.3.1 Circulating Tumor DNA (ctDNA)

ctDNA is one of the most extensively studied circulating biomarkers. It harbors genetic information such as mutations, methylation patterns, and viral DNA, thereby offering a promising approach to detecting minimal residual disease (MRD). A secondary analysis of the DART randomized controlled trial revealed that the persistence of HPV ctDNA (MRD‐positive) in plasma after surgery was significantly associated with a higher recurrence rate within 24 months, with a hazard ratio greater than five, underscoring ctDNA's high sensitivity for recurrence prediction [65].

Fakhry et al. demonstrated that ctDNA detection can identify recurrence earlier than traditional imaging methods in HPV‐positive oropharyngeal cancer patients, with significantly greater sensitivity and specificity. In their prospective study of 40 HPV‐positive patients, ctDNA was detected several months prior to tumor recurrence, thus offering the possibility for early intervention [40]. This study provides initial evidence supporting ctDNA's role in predicting recurrence in oropharyngeal cancer, confirming its utility as an early monitoring tool following treatment. Rettig et al. further validated ctDNA as a predictor of recurrence, showing that post‐treatment ctDNA detected in plasma was strongly correlated with relapse. Their study also demonstrated that ctDNA reflects tumor genetics and dynamically tracks disease progression in real time [66]. These findings solidified ctDNA's role in recurrence prediction, particularly in overcoming the limitations of traditional imaging methods. In addition, Lafata et al. expanded the role of ctDNA by exploring its use in monitoring recurrence post‐radiotherapy. Their research demonstrated that elevated ctDNA levels were closely associated with tumor metastasis and radioresistance, particularly in patients with local recurrence, further strengthening the association between ctDNA dynamics and recurrence [67]. Moreover, a multicenter study by Mielcarek‐Kuchta et al. affirmed the broad applicability and feasibility of ctDNA as a recurrence prediction tool [68]. The study showed that ctDNA could provide dynamic feedback at multiple post‐treatment time points, enabling real‐time monitoring of genetic changes in tumors and providing immediate insights into treatment responses. This reinforces the clinical relevance of ctDNA, indicating its effectiveness across different treatment regimens and underscoring its universal applicability. In conclusion, these studies collectively highlight ctDNA's substantial role in predicting recurrence in oropharyngeal cancer. ctDNA not only provides earlier recurrence signals than imaging techniques but also offers a reliable means of evaluating treatment response across various therapeutic approaches, thereby assisting clinicians in tailoring more personalized treatment plans. The scientific foundation laid by these studies supports the widespread application of ctDNA as a non‐invasive tool for recurrence prediction.

#### 3.3.2 Circulating Tumor Cells (CTCs)

Circulating tumor cells (CTCs) are tumor cells that have shed from primary or metastatic sites and entered the peripheral blood circulation. They are considered “seeds” of tumor metastasis and recurrence. The quantity and molecular characteristics of CTCs are closely linked to tumor biological behavior and clinical outcomes.

Numerous studies have highlighted the prognostic value of CTCs in head and neck squamous cell carcinoma (HNSCC). Zhang et al. (2024) conducted a study involving 154 HNSCC patients, where the detection of CTCs in baseline blood samples significantly increased the risks of recurrence and mortality. CTC positivity was identified as an independent predictor of progression‐free survival (PFS) and overall survival (OS) [69]. Similarly, Liu et al. (2020) reported that in patients with locally advanced HNSCC, a decrease in CTC levels following treatment was closely associated with improved PFS and OS, underscoring the potential of CTCs as a dynamic tool for monitoring treatment efficacy and predicting recurrence [70]. Furthermore, Zhang et al. (2022) found that baseline CTC counts were associated with treatment response and enabled personalized therapy and early prediction of recurrence risk [71]. In summary, CTCs, as biomarkers reflecting tumor metastatic potential and recurrence risk, can be used for early identification of high‐risk recurrence patients. They also demonstrate broad potential in dynamic efficacy monitoring and individualized treatment decision‐making.

#### 3.3.3 Exosomes and Non‐Coding RNAs

Exosomes are nanoscale vesicles (30–150 nm) secreted by cells into bodily fluids, capable of stably carrying various biomolecules such as miRNAs and lncRNAs. They play a role in intercellular communication and signal regulation. Due to the protection provided by their vesicle membrane, the nucleic acid components within exosomes are highly stable, making exosomes particularly advantageous in liquid biopsies and recurrence prediction.

In head and neck squamous cell carcinoma (HNSCC) research, circulating miRNAs have been confirmed to have significant prognostic value. Summerer et al. (2015) reported in the British Journal of Cancer that a group of plasma circulating miRNAs, such as miR‐142‐3p, miR‐186‐5p, and miR‐195‐5p, were significantly associated with progression‐free survival (PFS), local control rate (LRC), and overall survival (OS) in patients. These findings suggest that miRNAs could serve as potential biomarkers reflecting treatment response and recurrence risk [72]. Additionally, a systematic review and meta‐analysis by Qiu et al. further confirmed that several miRNAs, including miR‐21 and miR‐155, were significantly overexpressed in HNSCC. This abnormal expression pattern was closely associated with decreased survival rates and increased recurrence risk, indicating the clinical potential of miRNAs in recurrence prediction and prognosis evaluation [25]. In addition to miRNAs, exosomal lncRNAs are also attracting increasing attention. Certain lncRNAs can regulate the tumor immune microenvironment, promote immune evasion, and enhance angiogenesis, thereby indirectly increasing the risk of recurrence. While related studies in oropharyngeal cancer are still limited, existing evidence in head and neck cancers suggests that exosomal lncRNA abnormalities may be associated with poor prognosis. In conclusion, exosomes and their associated non‐coding RNAs, as circulating molecular biomarkers, offer the advantages of being non‐invasive, stable, and capable of dynamic monitoring, making them promising tools for predicting recurrence risk in oropharyngeal cancer. Future large‐scale, multi‐center clinical studies will be essential for validating these exosomal biomarkers, and they may ultimately be integrated into individualized follow‐up and treatment response evaluation systems.

#### 3.3.4 The Application of Multi‐Biomarker Combined Detection in Recurrence Prediction

Single biomarkers often have limitations in terms of sensitivity and specificity when used for recurrence prediction. In contrast, multi‐biomarker combined detection can integrate molecular information from various layers, offering a more comprehensive reflection of the tumor's biological characteristics.

In recent years, increasing efforts have been made to combine molecular biomarkers, protein markers, and circulating biomarkers to enhance the accuracy of recurrence prediction. For instance, in a clinical study by Chera et al. (2019), 103 HPV‐positive oropharyngeal cancer patients were monitored. The rapid clearance of plasma ctHPV16 DNA (a reduction of more than 95% within 28 days of chemoradiotherapy) was found to be strongly correlated with local disease control, while incomplete clearance was associated with a higher likelihood of recurrence [73]. This result suggests that dynamic detection of ctDNA could serve as a recurrence prediction tool, and when combined with other circulating biomarkers such as CTCs, the sensitivity and specificity of predictions would be further enhanced. Furthermore, a study by Rettig et al. (2019) demonstrated that combining HPV ctDNA with serum inflammatory protein markers (such as IL‐6 and CRP) could more accurately identify high‐risk patients post‐treatment, highlighting the complementary role of multidimensional molecular markers in recurrence prediction [74]. Additionally, integrative multi‐omics research provides a more solid foundation for the combined application of multiple biomarkers. Leemans et al. (2018) noted that integrating genomic, transcriptomic, and proteomic data enables the construction of multi‐layered models for predicting recurrence risk and guiding individualized follow‐up and treatment [75]. In summary, multi‐biomarker combined detection can address the limitations of single indicators, improving prediction accuracy while also providing new possibilities for precise follow‐up and individualized intervention. With the advancement of multi‐omics technologies and artificial intelligence methods, multi‐biomarker‐based predictive strategies are expected to become an important direction for managing oropharyngeal cancer recurrence in the future.

## 4 The Role of Biomarkers in Assessing Treatment Response

### 4.1 Prediction of Radiotherapy Response

Radiotherapy is a critical treatment modality for oropharyngeal cancer, especially in HPV‐positive patients, where its efficacy is notably significant. However, there is considerable individual variation in patient sensitivity to radiotherapy, with some patients still experiencing local recurrence or treatment resistance. Therefore, identifying reliable biomarkers to predict radiotherapy response is of vital clinical importance for optimizing treatment strategies and implementing personalized radiotherapy.

First, p16 protein serves as a classic surrogate marker for HPV‐related oropharyngeal cancer, with its overexpression reflecting Rb pathway inactivation caused by HPV integration. Numerous studies have shown that p16‐positive patients are generally more sensitive to radiotherapy and exhibit better prognosis. A prospective study by Lassen et al. (2014) found that p16 positivity was significantly associated with improved local control and overall survival rates [76]. Additionally, a systematic review and meta‐analysis by O'Rorke et al. (2012) indicated that HPV/p16‐positive patients had a nearly 50% reduction in overall survival risk, with significantly better progression‐free survival (PFS) and recurrence‐free survival rates compared to negative patients, further reinforcing the clinical value of p16 in predicting radiotherapy response [77]. In contrast, p53 protein aberrant expression is more common in HPV‐negative oropharyngeal cancers. TP53 mutations impair DNA damage repair and apoptosis pathways, leading to increased tumor cell survival post‐radiotherapy. Hong et al. (2016) reported that TP53 mutations were associated with shorter disease‐free and overall survival in over 200 oropharyngeal cancer patients, indicating a role for p53 alterations in radiotherapy resistance and recurrence [20]. Among signal transduction proteins, EGFR overexpression is considered one of the key mechanisms of radiotherapy resistance. Persistent EGFR activation promotes DNA damage repair and cell survival, thus impairing the apoptosis induced by radiotherapy. Clinical studies have shown that patients with high EGFR expression have a higher rate of local recurrence and poorer survival rates, making EGFR not only a predictor of recurrence but also a potential target for radiosensitization [42]. Furthermore, DNA repair‐related proteins have a direct impact on radiotherapy sensitivity. Polymorphisms or high expression of XRCC1 are associated with enhanced base excision repair capability, which may diminish the DNA damage effects induced by radiotherapy and increase the risk of recurrence. Early molecular epidemiological studies have pointed out that DNA repair gene polymorphisms play a crucial role in head and neck cancer susceptibility and treatment response [78]. A recent study by Coelho et al. (2024) found that the imbalance in MLH1/PMS2 expression was significantly associated with recurrence rates in non‐surgical OPSCC patients, particularly in p16‐positive tumors [79]. Although radiotherapy response and local recurrence were not directly evaluated, the study suggests a potential role for MLH1 in recurrence prediction. In addition, a meta‐analysis reported higher MLH1 promoter methylation in HNSCC patients than in healthy controls, although its association with radiotherapy sensitivity or recurrence remains unclear [80].

### 4.2 Prediction of Chemotherapy Response

Platinum‐based chemotherapy (e.g., cisplatin) remains a cornerstone in the treatment of locally advanced oropharyngeal cancer. However, therapeutic responses vary widely among patients, and a subset exhibits poor responsiveness or develops recurrence. Thus, identifying biomarkers predictive of chemotherapy sensitivity is crucial for optimizing treatment regimens and advancing personalized therapy.

DNA repair–related genes play a central role in mediating cisplatin sensitivity. Cisplatin kills tumor cells primarily by inducing DNA interstrand crosslinks; enhanced DNA repair capacity often leads to chemoresistance. Studies have associated genetic polymorphisms or elevated expression of XRCC1 and ERCC1 with resistance to platinum‐based regimens. For example, a meta‐analysis in head and neck squamous cell carcinoma (HNSCC) confirmed that the XRCC1 Arg399Gln polymorphism and high XRCC1 protein expression are significantly correlated with poorer survival outcomes, implying their potential in predicting chemotherapy response [59]. Detoxification and transport mechanisms are also pivotal in modulating chemotherapy efficacy. Polymorphisms in glutathione S‐transferase genes (e.g., GSTP1) may influence the detoxification of platinum compounds, thereby altering intracellular drug levels. Similarly, variants in transmembrane transporter genes such as ABCB1 or ABCG2 may promote drug efflux, reduce intracellular cisplatin accumulation, and contribute to resistance. Molecular pathways of resistance have also been elucidated. Recent studies in multiple cancer types highlight that exosomes and their miRNA cargo play significant roles in chemoresistance. For instance, exosomal miR‐21 has been implicated broadly in chemotherapy resistance across cancer types [81]. The mechanistic link is further supported by reviews showing how exosomal miRNAs modulate signaling networks (e.g., PTEN/PI3K, apoptosis pathways) to enable drug resistance [82]. In head and neck cancers, exosomal miRNAs are being actively investigated as non‐invasive biomarkers for prognosis and potentially treatment respons [83]. Circulating and exosomal biomarkers allow real‐time monitoring. In HPV‐associated oropharyngeal cancer, rapid clearance of plasma ctHPV16 DNA during chemoradiotherapy correlates with improved disease control and progression‐free survival, while persistent ctDNA indicates higher recurrence risk [73]. In summary, biomarkers predicting chemotherapy response in oropharyngeal cancer include DNA repair gene variants (XRCC1, ERCC1), detoxification and transport genes (GSTP1, ABCB1, ABCG2), and circulating/exosomal markers (ctDNA, exosomal miRNAs). Unlike signaling proteins associated with radiotherapy response (e.g., p16, p53, EGFR), these molecular signatures offer insights into chemoresistance mechanisms and support real‐time monitoring and precision chemotherapy.

### 4.3 Prediction of Immunotherapy Response

With the advent of immune checkpoint inhibitors (ICIs), immunotherapy has emerged as a key strategy for recurrent or metastatic oropharyngeal cancer. However, therapeutic efficacy varies markedly across patients, and only a subset derive sustained benefit. Hence, identifying robust predictive biomarkers is of critical clinical value.

PD‐L1 expression remains the most extensively applied biomarker for predicting the efficacy of ICIs. The KEYNOTE‐048 trial demonstrated that pembrolizumab, either as monotherapy or in combination with chemotherapy, significantly improved overall survival among patients with recurrent or metastatic head and neck squamous cell carcinoma (HNSCC), particularly those with a PD‐L1 combined positive score (CPS) ≥ 1, with the most pronounced benefit observed in patients with CPS ≥ 20 [84]. These findings further support that pembrolizumab monotherapy substantially prolongs survival in patients with PD‐L1 CPS ≥ 20 [84]. However, the predictive validity of PD‐L1 expression is not absolute. Variations in assay methodologies and scoring criteria can yield inconsistent results. A recent systematic review and meta‐analysis reported that high PD‐L1 expression on immune cells was significantly associated with improved overall and disease‐specific survival, whereas PD‐L1 expression confined to tumor cells did not demonstrate the same correlation [85]. This finding suggests that PD‐L1 expression within immune infiltrates may possess greater predictive value than tumor‐cell expression alone. A meta‐analysis of oropharyngeal squamous cell carcinoma (OPSCC) found that elevated PD‐L1 expression was associated with improved disease‐free survival but not overall survival, suggesting that its prognostic value may depend on assay methods and tumor subtype [86]. The immune microenvironment also exerts a profound influence on immunotherapeutic outcomes. HPV‐associated OPSCC is characteristically enriched with “inflamed” immune signatures, typified by abundant CD8^+^ and CD103^+^ T‐cell infiltration, which has been strongly linked to superior immunotherapy response and extended survival [87]. Furthermore, multicenter analyses have demonstrated that the T‐cell–inflamed gene expression profile (GEP) provides a more robust and independent predictor of ICI benefit and long‐term survival than PD‐L1 expression alone, underscoring the potential of transcriptomic immune‐activation signatures as more stable predictive biomarkers [88, 89]. Another emerging biomarker, tumor mutational burden (TMB), has shown predictive potential in multiple studies. Comprehensive analyses indicate that HNSCC patients with higher TMB benefit more from PD‐1 blockade and demonstrate superior survival outcomes. Nonetheless, the correlation between TMB and PD‐L1 expression remains inconsistent, suggesting that a multidimensional biomarker approach may be required to enhance predictive accuracy [90]. In summary, while PD‐L1 expression remains the most widely implemented clinical biomarker for predicting immunotherapy response, its limitations necessitate a more integrative framework. The incorporation of PD‐L1 expression (particularly within immune cells), immune‐cell infiltration patterns, T‐cell–inflamed GEP, TMB, and soluble PD‐L1 may enable the development of comprehensive predictive models, thereby facilitating more accurate response assessment and individualized optimization of immunotherapeutic regimens.

### 4.4 Evaluation of Combined Treatment Response

Combined therapies, such as chemoradiotherapy, immunochemotherapy, and immunotherapy combined with targeted therapies, represent critical treatment modalities for locally advanced and recurrent/metastatic oropharyngeal cancer. These approaches have shown significant improvements in overall survival and local control rates. However, clinical observations indicate that even with identical treatment regimens, substantial variability in patient responses remains, underscoring the need for reliable biomarkers to predict treatment efficacy and guide personalized therapy.

ctDNA clearance kinetics have emerged as an early predictive biomarker for assessing responses to chemoradiotherapy and immunotherapy combinations. In a multicenter study of HPV‐associated oropharyngeal cancer patients undergoing concurrent chemoradiotherapy, rapid clearance of ctHPV DNA in the early phase of treatment was associated with a significantly lower recurrence rate. In contrast, persistent ctDNA positivity indicated treatment resistance and poor prognosis [73]. These findings suggest that dynamic ctDNA monitoring provides real‐time molecular insights that can be crucial for evaluating the efficacy of combined therapies. The combined use of immune and molecular biomarkers is also gaining increasing attention. Economopoulou et al. (2019) reviewed that HPV DNA/E6/E7 antibodies, p16 expression, and PD‐L1 are evaluated as diagnostic and prognostic biomarker in head and neck squamous cell carcinoma (HNSCC). However, many of these biomarkers have yet to meet the necessary standards for sensitivity, specificity, reproducibility, and clinical applicability [91]. This suggests that multiplexed testing and multidimensional integration could be key strategies for enhancing predictive accuracy in the future. Moreover, the T cell–inflamed gene expression profile (GEP) has been shown to significantly correlate with benefits from immunotherapy. In an exploratory analysis of the KEYNOTE‐012 trial, this gene signature independently predicted treatment response, irrespective of PD‐L1 levels [92]. Genomic signatures of immune activity may serve as more reliable predictive tools. Serum markers of inflammation and angiogenesis, including elevated baseline VEGF, are associated with poorer outcomes in HNSCC, such as reduced event‐free survival and local/regional control [93]. Similarly, IL‐6 has been demonstrated to promote tumor cell proliferation and resistance to apoptosis through the JAK/STAT3 pathway, with high IL‐6 expression significantly correlating with increased recurrence risk and reduced overall survival [46]. Additionally, in HNSCC patients undergoing induction chemotherapy, dynamic changes in serum VEGF levels have been closely linked with treatment response and long‐term prognosis. These findings suggest that VEGF not only holds prognostic value prior to treatment but that its changes during therapy could serve as an important monitoring tool for assessing combined treatment efficacy and recurrence risk [94]. Proteins such as IL‐6 and VEGF reflect both the inflammatory and angiogenic status of the tumor microenvironment and could serve as supplementary tools for predicting the response to multimodal therapy, providing crucial insights for personalized combined treatment strategies. In summary, predicting the response to combined therapies should rely on a multidimensional biomarker approach, incorporating ctDNA dynamics, immune‐related markers (e.g., PD‐L1, TMB, GEP), and inflammation/angiogenesis factors. Future large‐scale, multi‐center prospective studies are needed to validate the clinical value of these biomarkers and to explore integrated composite predictive models, thereby enhancing the precision and personalization of combined treatment strategies.

## 5 Clinical Applications and Challenges of Biomarkers

Biomarkers have demonstrated tremendous potential in predicting recurrence and evaluating treatment responses in oropharyngeal cancer. However, their clinical application remains hindered by several challenges. In recent years, with the advancement of liquid biopsy technologies, biomarkers such as ctDNA, circulating tumor cells (CTCs), and exosomes have gradually entered the clinical exploration phase, providing non‐invasive, dynamically traceable methods for early monitoring of recurrence and assessing treatment efficacy [73]. Simultaneously, certain histological biomarkers have achieved clinical translation, including PD‐L1 testing for immunotherapy stratification and p16 status as a routine prognostic tool in oropharyngeal cancer [95]. These advancements suggest that certain biomarkers are progressing from laboratory research to clinical application. Nonetheless, several limitations persist. First, significant discrepancies in detection platforms and analytical methods across different research centers have led to a lack of comparability and reproducibility of results [91]. For instance, PD‐L1 testing often exhibits inconsistencies due to variations in antibody types, scoring methods, and expression sites, limiting its clinical applicability. Second, the sensitivity and specificity of most biomarkers are insufficient to directly guide clinical decision‐making. In particular, in early recurrence risk prediction, false‐negative and false‐positive rates remain high [75]. Moreover, the dynamic changes in biomarker levels and their relationship with tumor heterogeneity are complex, and there is currently no unified standard for monitoring time points and threshold values [87].

Future research must rely on large‐scale, multi‐center prospective validation studies to ensure the stability and clinical generalizability of biomarkers. Additionally, the integration of multi‐omics approaches (genomics, proteomics, immunomics, etc.) with artificial intelligence (AI) analysis holds the potential to overcome the limitations of single biomarker predictive capabilities, facilitating the development of composite predictive models and enabling more precise assessments of recurrence risk and treatment response [96]. In conclusion, although biomarkers have shown promise in clinical applications for oropharyngeal cancer, their broader adoption is limited by factors such as inconsistent detection methods, variable sensitivity and specificity, and a lack of large‐scale validation. Future efforts should focus on multi‐biomarker panels and multicenter clinical trials to enable their full integration into clinical practice and to support precision diagnosis and treatment.

## 6 Future Directions and Research Prospects

As molecular medicine and precision oncology continue to advance, the potential applications of biomarkers in predicting recurrence and evaluating treatment responses in oropharyngeal cancer are expanding rapidly. Future research will likely focus on the discovery of novel biomarkers, the integration of multi‐omics and artificial intelligence, and the exploration of personalized treatment strategies.

Although traditional molecular, protein, and circulating biomarkers have shown promise, they remain insufficient to fully capture the complex biology of oropharyngeal cancer. With the continued development of multi‐omics technologies, including genomics, transcriptomics, proteomics, and metabolomics, the integration of these diverse data types is poised to uncover new candidate biomarkers. For instance, multi‐omics analyses that combine genomic and transcriptomic data have already identified distinct molecular subtypes of head and neck squamous cell carcinoma (HNSCC) and revealed differences in their treatment sensitivities [97]. Furthermore, recent studies have leveraged multi‐cohort data combined with machine learning to build prognosis models based on multi‐omics, significantly enhancing the accuracy of recurrence predictions [98]. These advances suggest that integrating omics approaches will be a critical avenue for future biomarker research.

Artificial intelligence (AI) and big data technologies also show tremendous promise in the field of head and neck oncology. AI's ability to perform pattern recognition and predictive modeling on large‐scale biomarker data enhances the precision of recurrence risk and treatment response assessments. Recent reviews have highlighted that AI applications in head and neck cancer research now extend beyond radiomics and pathology to include multimodal integration of liquid biopsy data, with the potential to drive the clinical implementation of composite predictive models [99]. In parallel, big data‐driven research frameworks are accelerating the discovery and validation of novel biomarkers, enabling them to be dynamically updated and applied in real‐time. Looking ahead, personalized management will emerge as the core direction for clinical translation. Risk stratification based on biomarkers not only aids in identifying high‐risk patients but also provides a foundation for tailoring individualized treatment plans. For example, patients with persistently positive ctDNA could undergo more intensive follow‐up or receive additional adjuvant therapy post‐treatment, while those with high PD‐L1 expression may benefit more from immune checkpoint inhibitors [100]. However, for these applications to achieve full clinical integration, large‐scale, multi‐center prospective clinical trials are essential, alongside the establishment of standardized testing and interpretation guidelines to ensure the robustness and applicability of results. In summary, future research should focus on identifying novel biomarkers, integrating multi‐omics with AI, and refining personalized treatment strategies. Supported by interdisciplinary collaboration and clinical big data, biomarkers are set to play an increasingly central role in predicting recurrence and evaluating treatment responses in oropharyngeal cancer.

## 7 Conclusion

Over the past decade, biomarker research in oropharyngeal cancer has advanced considerably, improving the ability to estimate recurrence risk and evaluate treatment response. Investigations have covered diverse biological layers, including genomic and epigenomic changes, protein signaling networks, and apoptosis‐associated indicators. At the same time, the development of minimally invasive approaches—particularly those based on circulating analytes such as ctDNA, circulating tumor cells, exosomes, and microRNAs—has expanded opportunities for real‐time disease monitoring. Together, these biomarker strategies have strengthened clinical risk stratification, supported response assessment, and facilitated more individualized patient management. Among currently available candidates, p16 status, PD‐L1 expression, and circulating tumor HPV DNA‐based assays stand out as the most clinically mature options, with clear potential for near‐term use in both surveillance and treatment guidance. Notably, p16 and PD‐L1 testing has already been incorporated into routine practice and has become integral to patient stratification and therapeutic decision‐making.

Nevertheless, several issues continue to limit broader clinical adoption. Substantial heterogeneity in assay platforms, laboratory procedures, and analytical pipelines across studies undermines cross‐study comparability and reproducibility. In addition, many proposed biomarkers demonstrate variable diagnostic performance across cohorts, with sensitivity and specificity that remain insufficient for independent clinical decision‐making. Progress is also constrained by the lack of standardized cutoff values, the limited number of large prospective validation studies, and the incomplete integration of biomarker‐driven algorithms into established clinical workflows. These challenges are further intensified by intratumoral heterogeneity and by dynamic shifts in biomarker profiles over the course of disease progression and treatment.

Looking forward, further progress will likely depend on integrating multi‐omics profiling with computational approaches and well‐curated, large‐scale clinical datasets. Rather than relying on single biomarkers, composite panels that incorporate molecular, immune, and circulating features may provide more robust and clinically actionable prediction models. With multicenter prospective validation and harmonized protocols for biomarker measurement and interpretation, these approaches are expected to move more effectively into routine clinical practice, enabling more precise surveillance and more personalized treatment strategies for patients with oropharyngeal cancer.

## Author Contributions

Yunxia Chen and Wenyu Zhang should be considered joint first authors. Zhihao Xie and Ping Zhou contributed equally as corresponding authors. Yunxia Chen: Conceptualization, Methodology, Resources, Writing – original draft. Wenyu Zhang: Conceptualization, Investigation, Methodology, Validation, Supervision. Xiang Gao: Resources, Visualization, Methodology, Validation, Formal analysis, Data curation. Kongling Xing: Methodology, Validation. Yijing Ren: Methodology, Validation. Jinyu Hu: Methodology, Validation. Zhihao Xie: Investigation, Supervision, Writing – review and editing. Ping Zhou: Methodology, Supervision, Writing – review and editing, Funding acquisition. All authors read and approved the final manuscript.

## Funding

This work was supported by Hainan Province Science and Technology Special Fund (ZDYF2022SHFZ132, ZDYF2024SHFZ045), National Natural Science Foundation of China (82260474, 82303935), and the Scientific Research Project of Health and Family Planning Industry in Hainan Province, China (22A200068).

## Disclosure

We confirm that all scientific content, data interpretation, and conclusions in this manuscript were independently generated by the authors. AI‐assisted tools were used solely for language polishing and grammatical refinement. No AI was involved in data analysis, interpretation, or manuscript conceptualization.

## Conflicts of Interest

The authors declare no conflicts of interest.

## Data Availability

No new data were created or analyzed in this study. Data sharing is not applicable to this article.
